# Elevated IgG4 serum levels in patients with cystic fibrosis

**DOI:** 10.1371/journal.pone.0181888

**Published:** 2017-09-18

**Authors:** Axelle Clerc, Quitterie Reynaud, Stéphane Durupt, Colette Chapuis-Cellier, Raphaële Nové-Josserand, Isabelle Durieu, Jean Christophe Lega

**Affiliations:** 1 Département de médecine interne et Centre de Référence Mucoviscidose, Centre Hospitalier Lyon Sud, Hospices Civils de Lyon, Pierre Bénite, France; 2 EA Health Services and Performance Research (*HESPER*) 7425, Lyon, France; 3 Université Claude Bernard Lyon 1, Lyon, France; 4 Département d’immunologie, Centre Hospitalier Lyon Sud, Hospices Civils de Lyon, Pierre Bénite, France; 5 UMR 5558, Laboratoire de Biométrie et Biologie Evolutive, Equipe évaluation et Modélisation des Essais Thérapeutiques, Claude Bernard University of Lyon 1, Lyon, France; Laurentian, CANADA

## Abstract

**Objective:**

Serum immunoglobulin (Ig) G4 elevation has been associated with several pathological conditions other than IgG4-related disease (IgG4-RD). In cystic fibrosis (CF), an elevation of specific IgG4 has been associated with colonization and infection by *Pseudomonas aeruginosa*. IgG4 elevation may be a marker of chronic infection or inflammatory stimulation. The aim of this study was to explore the prevalence of elevated IgG4 levels in CF and its correlation with the major clinical and microbiological features found in CF patients.

**Methods:**

In a cross-sectional study, we analyzed data from a large cohort of adult CF patients attending the CF center of Lyon University Hospital. An elevated IgG4 level was defined as being above the cut-off value of 135 mg/dL.

**Results:**

One hundred and sixty-five CF patients were analyzed. An IgG4 elevation was detected in 43 patients (26%). Compared with the control group (≤ 135 mg/dL), high IgG4 patients exhibited a greater prevalence of *Staphylococcus aureus* colonization and higher IgG, IgG1, IgG2 and IgE levels. No significant differences were observed in terms of pulmonary function, colonization with *Pseudomonas aeruginosa*, or the annual rate of bronchial exacerbations.

**Conclusion:**

An elevated IgG4 serum level was frequently detected in adult CF patients and did not appear to be associated with poor lung function. We suggest that IgG4 elevation is a marker of the activation of tolerance. Its clinical significance remains to be demonstrated.

## Introduction

Cystic fibrosis (CF) is the most common severe autosomal recessive genetic disease in Europe. The disease is caused by a mutation in the gene encoding the cystic fibrosis transmembrane conductance regulator protein, a chloride ion channel whose abnormal function leads to the hyperviscosity of exocrine secretions and obstructive damage to many organs, including the lung, pancreas, hepatobiliary tracts, and vas deferens [1]. Immunoglobulin (Ig) G4 is a unique antibody in both structure and function. This molecule accounts for less than 5% of the total IgG in healthy persons and is the least abundant IgG subclass. The normal range of serum IgG4 concentrations is 24–135 mg/dL, and IgG4 concentrations within individuals are generally stable. Unlike other IgG subclasses, IgG4 is unable to fix complement or precipitate antigens [2,3]. Despite its traditional role as an antiinflammatory immunoglobulin, IgG4 is presumed to play a central role in certain immune-mediated conditions.

The attention to serum IgG4 concentrations has increased in recent years due to the emerging concept of IgG4-related disease (IgG4-RD), an auto-immune disease leading to the tumor-like swelling of involved organs, lymphoplasmacytic infiltrate enriched in IgG4-positive plasma cells, and variable degrees of fibrosis. Furthermore, serum IgG4 elevation has been associated with several pathological conditions other than IgG4-RD [4,5]. Ebbo et al. reported elevated levels of serum IgG4 (> 135 mg/dL) in a series of 59 patients. Only 6 of the patients presented with IgG4-RD; the other primary diagnoses included repeated infections (n = 15), autoimmune diseases (n = 8), cancers (n = 4), primary immune deficiencies (n = 8), idiopathic interstitial pneumonitis (n = 3), and vasculitis (n = 2). Two patients presented with CF [4]. Yun et al described 61 patients with IgG4 concentrations greater than 130 mg/dL. Nine of these patients had convincing clinical features of IgG4-RD. In this cohort, 16 patients with elevated serum IgG4 concentrations had respiratory diseases, in particular bronchiectasis, and 5 were CF patients [5]. These 2 studies confirmed that a serum IgG4 elevation above 135 mg/dL is not a specific marker for the diagnosis of IgG4-RD and can be observed in several clinical conditions, among them CF. In a small series of 16 patients published in 1976, Shakib et al showed that 44% of CF patients had elevated IgG4 concentrations [6]. Factors associated with IgG4 elevation in CF have already been largely studied, and the role of chronic colonization with *Pseudomonas aeruginosa* has remained unclear [7–11].

Many studies have evaluated the immunologic profile and IgG levels in CF patients and their correlations with clinical parameters. However, none have analyzed IgG4 levels as a putative marker of chronic infection or inflammatory stimulation leading to specific symptoms and organ dysfunction. In this study, we aimed to report the prevalence of patients with serum IgG4 elevation in CF and the clinical and microbiological features associated with this marker.

## Patients and methods

### Study population

All consecutive adult CF patients with an available measure of serum protein electrophoresis and an IgG4 level obtained during a routine annual visit to our CF center were included in a prospective cohort. CF patients with lung or liver transplantation were excluded in order to avoid bias related to immunosuppressive therapies. This study was approved by our local Ethical Committee (Hospices Civils de Lyon) who waived the need for participants consents. Rrecords were accessed anonymously.

### Clinical data

Data were prospectively collected from medical records between June 2013 and June 2014 during routine annual clinic visits under stable clinical conditions that included a sputum sample collected for bacterial and fungal examination. The clinical data extracted included the patient’s age, weight, height, body mass index (BMI), CF genotype, pulmonary function (forced expiratory volume in one second [FEV_1_]), presence of allergic bronchopulmonary aspergillosis (ABPA), number of annual exacerbations, bacteriological chronic colonization (identification of the same microorganism during over a period of 6 months in three samplings), the results from sputum collection, digestive clinical status (hepatic cirrhosis, ursodeoxycholic acid treatment, exocrine pancreatic insufficiency, distal intestinal obstruction syndrome), CFTR-related diabetes, glycosylated hemoglobin levels (HbA1c), osteoporosis, and characteristics and presence of arthralgias. Allergic bronchopulmonary aspergillosis was defined by elevated serum IgE levels (> 1000 IU/mL, unless the patient was receiving corticosteroid therapy), aspergillus specific IgE (> 500 IU/mL) and/or IgG, and recent abnormalities on chest radiography.

### Immunoglobulin level quantification

Immunologic parameters (serum protein electrophoresis, IgG subclasses, and IgE) were recorded. Total serum IgG, IgA and IgM serum levels were measured by immunonephelometric assay (BN ProSpec^®^ Siemens). Normal ranges were 682–1266 mg/dL for serum IgG, 80–254 mg/dL for IgA, 71–201 mg/dL for IgM. Subclasses of IgG levels were determined by immunoturbidimetry (SPA Plus^®^ The Binding Site). The normal ranges were 382.4–928.6 mg/dL for IgG1, 241.8–700.3 mg/dL for IgG2, 21.8–176 mg/dL for IgG3, and 3.9–86.4 mg/dL for IgG4. The patients were divided into either the IgG4high group (IgG4 > 135 mg/dL) or the IgG4norm group (IgG4 ≤ 135 mg/dL) according to their IgG4 levels. The patients’ serum IgE levels were determined by a fluorescence enzyme immunoassay (ImmunoCAP 250^®^ ThermoFisher), with a normal range < 150 UI/mL.

### Statistical analysis

Data were presented as the means and the standard deviations for the continuous variables. Fisher’s exact test and the chi-squared test were used to compare the categorical variables. The Mann-Whitney test or Student’s t-test were used to analyze the continuous variables. The correlation between Ig subtypes was assessed by the Pearson correlation coefficient. P values less than 0.05 were considered statistically significant.

## Results

### Patient characteristics

One hundred and sixty-five CF patients (80 women; mean patient age of 29 years ± 9 years) attending the Lyon University adult CF center were included during the one-year period.

According to our definition, an IgG4 elevation was detected in 43 (26.1%) of the patients. The mean serum IgG4 level in the IgG4high group was 228 mg/dL ± 68.7 mg/dL. The mean age and BMI were not significantly different between the IgG4high and IgG4norm groups (Table 1 and S1 Table).

**Table 1:** Characteristics of patients with elevated and normal IgG4 levels.

	IgG4high group(n = 43)	IgG4norm group(n = 122)	p
Male sex (%)	28 (65)	57 (47)	0.051
Age, years (mean ±SD)	29.7 ±8.7	28.7 ±9.1	0.57
F508del homozygosis (%)	20 (47)	51 (42)	0.60
Diabetes (%)	11 (26)	29 (24)	0.84
Insulin therapy (%)	6 (14)	37 (30)	0.81
Osteoporosis (%)	2 (5)	10 (8)	0.73
Arthralgia (%)	3 (7)	6 (5)	0.70
Biliary lithiasis (%)	12 (28)	50 (41)	0.15
Liver cirrhosis (%)	2 (5)	3 (3)	0.61
Distal intestinal obstruction syndrome (%)	7 (16)	20 (16)	1.00
Exocrine pancreatic insufficiency (%)	42 (98)	109 (89)	0.11
BMI, kg/m^2^ (mean ±SD)	20.6 ±2.6	20.5 ±2.6	0.98
IgG4, mg/dL (mean ±SD)	228 ±69	55 ±34	<0.0001
IgG4, mg/dL (median, range)	218 (138–406)	49 (5–135)	<0.0001
IgG, mg/dL (mean ±SD)	1611 ±406	1393 ±351	<0.0001
IgG1, mg/dL (mean ±SD)	814 ±280	676 ±208	0.001
IgG2, mg/dL (mean ±SD)	679 ±191	493 ±199	<0.0001
IgG3, mg/dL (mean ±SD)	104 ±851	85 ±55	0.21
IgE, IU/mL (mean ±SD)	643 ±761	189 ±345	0.03

Ig: immunoglobulin; *CFTR*: Cystic Fibrosis Transmembrane Conductance Regulator; BMI: body mass index; FEV_1_: forced expiratory volume in one second; SD: standard deviation.

### Association of IgG4 levels with clinical and laboratory manifestations

#### Respiratory manifestations

The mean FEV_1_ was similar between the two groups (63.2% ± 25.3% in the IgG4high group and 63% ± 23.4% in the IgG4norm group). The only difference observed between the 2 groups was the prevalence of *Staphylococcus aureus* (*Sa*), which was higher in the IgG4high patients than in the IgG4norm patients (83% versus 65%, p = 0.02). No statistically significant difference were observed for methicillin resistant *Staphylococcus aureus* (MRSA), *Pseudomonas aeruginosa*, other non-fermenters (*Burkholderia* spp, *Stenotrophomonas* spp), nontuberculous *Mycobacterium*, *Aspergillus fumigatus* colonization, ABPA and the number of annual exacerbations. Despite no difference in terms of *Aspergillus fumigatus* colonization and ABPA, the mean IgE level was 463 ±116 UI/mL in the IgG4high group versus 189 ±31 UI/mL in the IgG4norm group (p = 0.002). None of the patients in the IgG4high group had chronic respiratory insufficiency. Details are shown in Table 2. The IgG4 levels were not predictive of chronic respiratory failure, long-term oxygen therapy or non-invasive ventilation at home.

**Table 2:** Respiratory characteristics of patients with elevated and normal IgG4 levels.

	IgG4high group(n = 43)	IgG4norm group(n = 122)	P
FEV_1_, % (mean ±SD)	63.2 ±25.3	63.0 ±23.4	0.97
Bronchial colonization (%)			
Staphylococcus aureus	35 (83)	75 (65)	0.02
MRSA	8 (19)	12 (10)	0.17
Pseudomonas aeruginosa	26 (60)	87 (71)	0.25
Other non-fermenter^a^	6 (14)	17 (14)	1.00
Non tuberculous mycobacteria	2 (5)	7 (6)	1.00
Aspergillus fumigatus colonization	11 (26)	30 (25)	1.00
Number of annual exacerbations (mean ±SD)	1.2 ±1.5	1 ±1.4	0.66
Total IgE, IU/mL (mean ±SD)	463 ±116	189 ±31	0.002
ABPA (%)	7 (16.3)	18 (14.8)	NS

IgG4: immunoglobulin G4; MRSA: methicillin resistant *Staphylococcus aureus*; IgE: immunoglobulin E; ABPA: allergic bronchopulmonary aspergillosis.

*NS: not significant*.

^a^*Burkholderia* spp, *Stenotrophomonas* spp.

#### Extra-respiratory manifestations

The prevalence of diabetes, treatment with insulin, and HbA1c values did not differ between the two groups. A majority of the patients had received supplementation for exocrine pancreatic insufficiency since childhood; there was not an observed difference between the two groups. No difference in the occurrence of cirrhosis, requirement of ursodeoxycholic acid treatment, or number of distal intestinal obstruction syndromes was noted. The occurrence of arthralgia did not differ between the 2 groups. The presence of osteoporosis was not correlated to the patients’ IgG4 levels.

### Correlation between IgG4 value and other Ig

**Fig 1:**
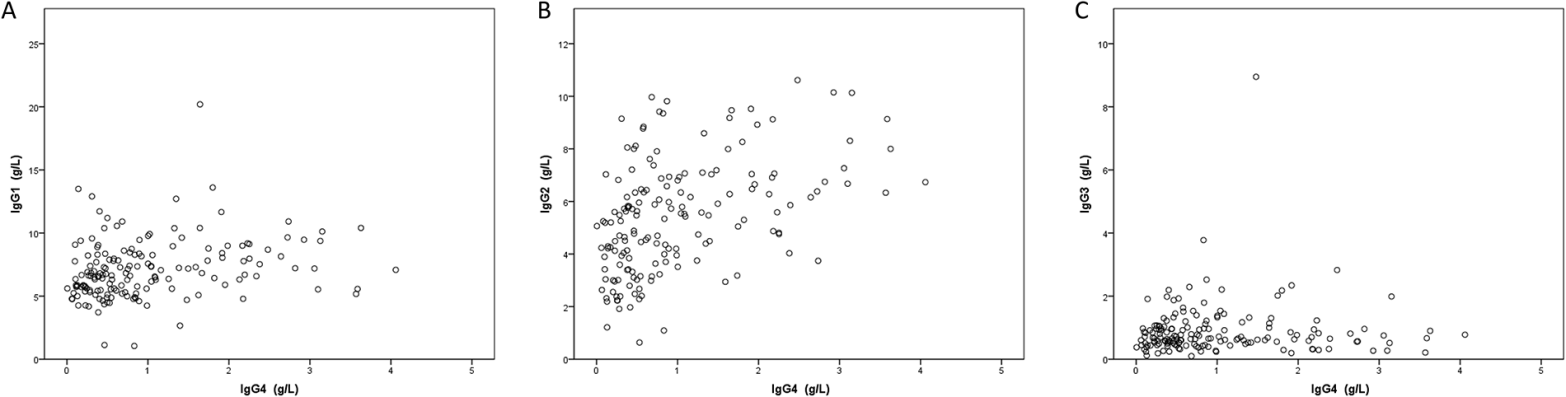
Scatter plot of serum IgG1 (A), IgG2 (B), and IgG3 (C) levels according to IgG4 elevated levels.

**Fig 2:**
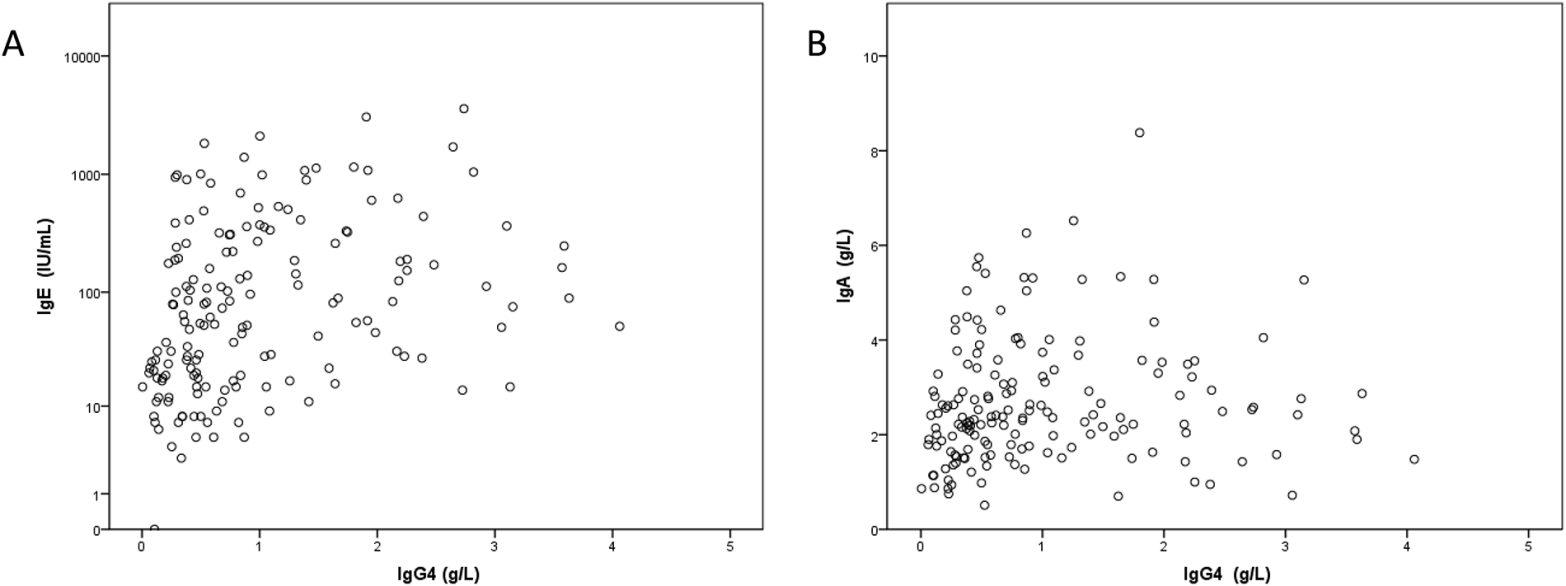
Scatter plot of serum IgE (A) and IgA (B) according to IgG4 elevated levels.

**Fig 3:**
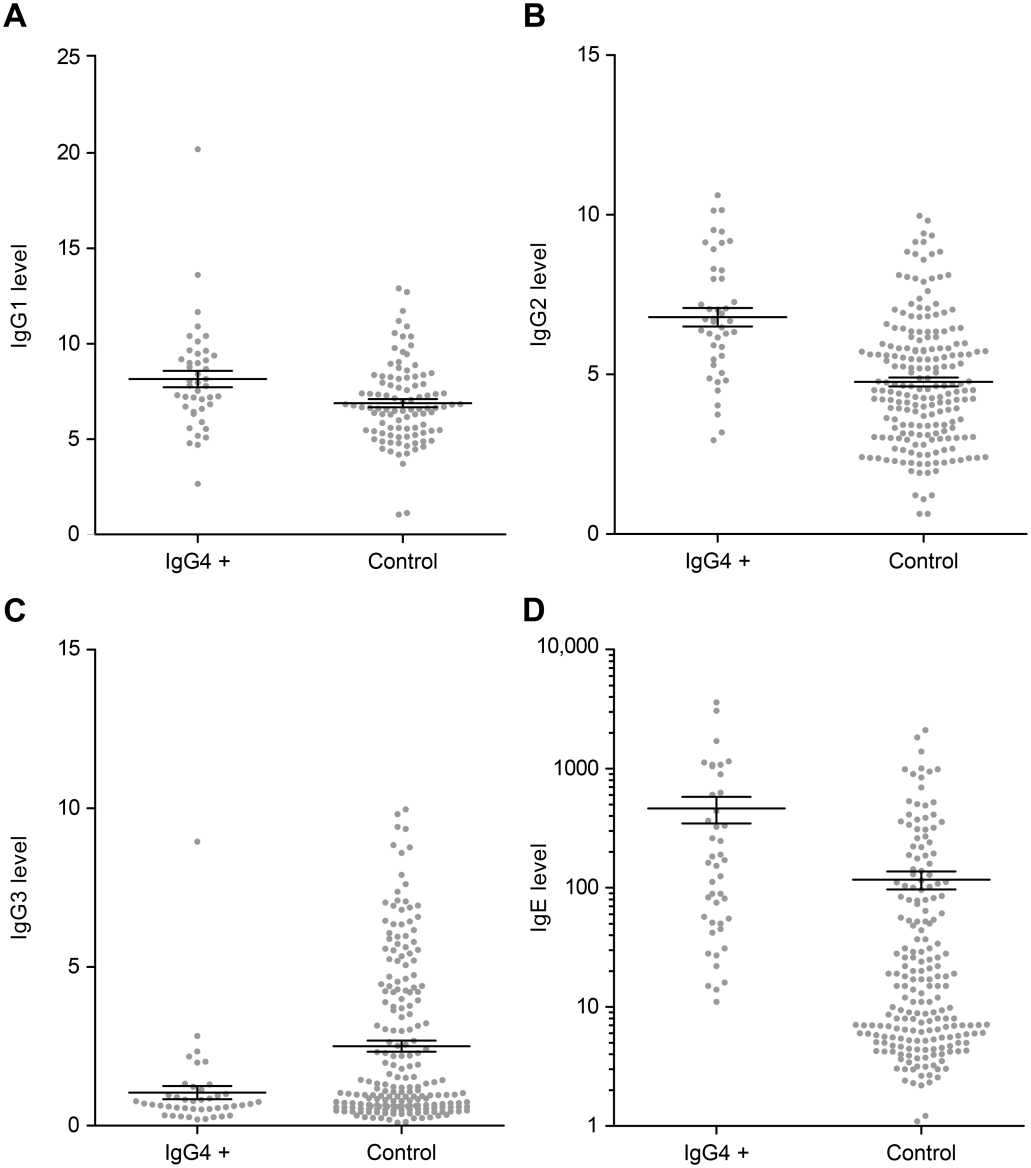
Serum IgG1, IgG2, and IgE levels in patients with IgG4 elevated levels (IgG4high group: IgG4 > 135 mg/dL) versus control. A: Serum IgG1 (mg/dL) in patients with elevated IgG4 levels versus controls; B: Serum IgG2 (mg/dL) in patients with elevated IgG4 levels versus controls; C: Serum IgG3 (mg/dL) in patients with elevated IgG4 level versus controls; D: Serum IgE (IU/mL) in patients with elevated IgG4 levels versus controls (Ig: immunoglobulin).

The elevation of IgG4 was strongly associated with hypergammaglobulinemia (p = 0.0001) (Table 3). The IgG4 level was correlated with IgG1 (p = 0.001 by Pearson’ correlation), IgG2 (p <0.0001 by Pearson’ correlation), IgE (p <0.0001 by Pearson’ correlation), IgA (p = 0.003 by Pearson’ correlation) but not to IgG3 (p = 0.99 by Pearson’ correlation) “Figs 1, 2 and 3” (Table 3). In the IgG4high group compared to control, The IgG1 level (mean concentration 8.1 ± 2.8 g/L versus 6.8 ± 2.1 g/L, p = 0.001), IgG2 level (mean concentration 6.8 ± 1.9 g/L versus 4.9 ± 2.0 g/L, p <0.0001), IgE level (mean concentration 643 ± 761 IU/mL versus 189 ± 345 IU/mL, p = 0.03), but not IgG3 level (mean concentration 1.0 ± 0.8 g/L versus 0.9 ± 0.6 g/L, p = 0.21) were significantly higher “Fig 3”.

**Table 3:** Relation between IgG4 and other immunoglobulins by Pearson’ correlation.

	**IgG**	**IgG4**	**IgG1**	**IgG2**	**IgG3**	**IgA**	**IgE**
IgG	1	0.393p<0.0001	0.73p<0.0001	0.677p<0.0001	0.247p = 0.001	0.375p<0.0001	0.1150.14
IgG4		1	0.25p = 0.001	0.459p<0.0001	0.043p = 0.58	0.075p = 0.34	0.228p = 0.003
IgG1			1	0.292p<0.0001	-.004p = 0.96	0.267p = 0.001	0.130p = 0.1
IgG2				1	0.224p = 0.004	0.265p = 0.001	0.054p = 0.49
IgG3					1	0.166p = 0.03	0.162p = 0.04
IgA						1	0.129p = 0.003
IgE							1

## Discussion

To our knowledge, this study is the first to report the frequency of high IgG4 values in a large cohort of adult CF patients. In our CF cohort, the prevalence of IgG4 values > 135 mg/dL was 26%. This study did not suggest an association between the elevation of serum IgG4 levels and the clinical features evocative of an inflammatory pattern similar to that of IgG4normRD. Our results showed an association between high IgG4 value and *Staphylococcus aureus* colonization and between hypergammaglobulinemia and high IgG1, IgG2, and IgE values.

Several studies have evaluated the immunologic profile and IgG in pediatric CF patients [12–15]. These publications reported a relationship between hypergammaglobulinemia and a decline in FEV_1_, whereas hypogammaglobulinemia was associated with stable pulmonary function and a better prognosis. Only one study included adult CF patients [16]. In this study, Levy et al demonstrated a correlation between higher IgG levels and clinical disease severity. In patients with elevated IgG levels and a lower FEV_1_, the IgG levels may represent a hyperimmune response that is ineffective against infection and possibly destructive of the airway. Overall, an elevated serum IgG level is clearly a marker of an increased inflammatory response to pathogens, and its correlation with lung function in CF patients supports the link between inflammation and deterioration in respiratory function in CF. In our study, we demonstrated that an elevation in serum IgG4 levels was associated with hypergammaglobulinemia, particularly the elevation of IgG1 and IgG2. Nevertheless, we did not find a correlation between a decline in FEV_1_ and an elevation in the IgG4 levels. Thus, our results suggest a neutral effect of IgG4 as an anti-inflammatory immunoglobulin in patients with hypergammaglobulinemia and without the progression of pulmonary disease. Furthermore, these results suggest that hypergammaglobulinemia may be promoted by a non-specific immune activation, which would explain the high frequency of autoantibodies in adult CF patients reported in our group [17].

In our study, we highlighted the association between elevated IgG4 values and *Staphylococcus aureus* colonization. There was no association with MRSA or other types of bacterial colonization (*Burkholderia* spp, *Stenotrophomonas* spp, *Pseudomonas aeruginosa*) or with the number of exacerbations. *Staphylococcus aureus* colonization in the adult CF patients was frequent, and as high as 83% in the IgG4high group versus 65% in the IgG4norm group. It is the most common species colonizing the CF respiratory tract. Moreover, chronic *Pseudomonas aeruginosa* pulmonary infection is associated with a decline in lung function, increased exacerbations and reduced survival in people with CF [18,19]. *Pseudomonas aeruginosa* can induce an inflammatory response that leads to tissue breakdown and severe lung damage. Many studies have analyzed the inflammatory biomarkers in the sputum and bloodstream of CF patients to characterize pulmonary inflammation [20,21]. In our study, we found no association with *Pseudomonas aeruginosa* colonization, as would have been expected to increase in the group with hypergammaglobulinemia [12,15,22,23]. Finally, elevated IgG4 levels may have protective and anti-inflammatory effects in the evolution of the disease.

Our study addressed the association between the elevation of IgG4 levels and with IgE levels. Our results are in accordance with the literature and can be explained by a common regulation of IgE and IgG4 that is dependent on Th2 cells [2]. IgG4 antibodies are produced largely after prolonged immunization. In immunoallergology, the levels of allergen-specific IgG4 and IgE are clinical markers used to detect the efficacy of tolerance-inducing therapies in patient with allergies, but this method of detection depends on the specific rather than the total IgG4 and IgE. The presence of allergen-specific IgG4 indicates that tolerance-inducing mechanisms have been activated. It may play a role in allergic disorders by competing for allergens with the IgE bound to mast cells, thus blocking their stimulation and inhibiting IgE-mediated hypersensibility [24]. In this analysis, we did not observe any relationship between the IgG4 levels and hypersensibility manifestations or ABPA.

Our study suffered from several limitations. First, this study was conducted over one year and the evolution of the IgG4 levels was not assessed. Second, our hypothesis was not supported by experimental evidence illustrating the relation between IgG4 levels and a putative anti-inflammatory effect in CF. Third, our observations need to be interpreted with caution because of the risk of the inflation of false positive tests related to multiple comparisons.

## Conclusion

In conclusion, high IgG4 serum values were detected in 26% of the adult CF patients. We found an association between high IgG4 values and *Staphylococcus aureus* colonization; hypergammaglobulinemia; and IgG1, IgG2, and IgE concentrations. High IgG4 values were not associated with lung function decline. The elevation of IgG4 levels may be a consequence of chronic B-cell stimulation of gammaglobulin production associated with the activation of tolerance induction. Further studies are needed to understand the clinical significance of high IgG4 values in CF.

## Supporting information

S1 Table IGG42016. (XLSX)

## Abbreviations

ABPA: allergic bronchopulmonary aspergillosis
BMI: body mass index
CF: cystic fibrosis
*CFTR*: cystic fibrosis transmembrane conductance regulator
FEV_1_: forced expiratory volume in one second
HbA1c: glycosylated hemoglobin levels
Ig: immunoglobulin
IgG4-RD: immunoglobulin G4-related disease
MRSA: methicillin resistant *Staphylococcus aureus*
NS: not significant
